# Exosomal miR‐27a‐5p Helps Differentiate Parathyroid Carcinoma From Adenoma and Inhibits Apoptosis in Parathyroid Carcinoma Cells

**DOI:** 10.1155/ije/5562632

**Published:** 2026-06-08

**Authors:** Jiacheng Wang, Yuting Wang, Qian Wang, Teng Zhao, Hong Shen, Xing Liu, Dalin Feng, Rongfang Shen, Li Zhao, Jian Huang, Wenjing Yang, Bojun Wei

**Affiliations:** ^1^ Department of Thyroid and Neck Surgery, Beijing Chaoyang Hospital Affiliated to Capital Medical University, Beijing, 100020, China, bjcyh.com.cn

**Keywords:** apoptosis, exosome, MicroRNA, parathyroid carcinoma, primary cell culture

## Abstract

**Purpose:**

The differential diagnosis of parathyroid carcinoma (PC) or parathyroid adenoma (PA) in parathyroid tumors is crucial for their treatment and prognostic outcomes. We have reported previously that exosomal miR‐27a‐5p is different in PC or PA, although there are no published studies on a large number of samples, with the role of miR‐27a‐5p in regulating the effects of PC progression remaining unknown. The current study was designed to investigate the differential expression pattern of exosomal miR‐27a‐5p in both PC and PA and its relationship with clinicopathologic features, with the aim of further investigating the effect of PC at the cellular level.

**Methods:**

The miRNA profiles of serum exosomes from 9 PC and 9 PA patients were evaluated using high‐throughput sequencing. The differential expression of exosomal miR‐27a‐5p was detected in 29 PC and 36 PA patients. We also evaluated the diagnostic efficacy of exosomal miR‐27a‐5p to identify PC or PA and examined its correlation with the clinicopathologic features of PC. Primary PC cells were obtained by primary cell culture, while the effect of exosomes on PC cell apoptosis was investigated using miR‐27a‐5p mimic transfection and exosome mixed culture. Finally, the possible target gene of miR‐27a‐5p, PIK3CA, was identified using a dual‐luciferase assay.

**Results:**

Exosomal miR‐27a‐5p differed significantly in PC and PA and was upregulated in PC with good diagnostic efficacy. Exosomal miR‐27a‐5p was also associated with T‐staging of PC. miR‐27a‐5p inhibited PC cell apoptosis, an action that may be related to its target gene PIK3CA. However, the specific mechanism of this inhibition requires further exploration.

**Conclusions:**

The exosomal miR‐27a‐5p assay is a noninvasive liquid biopsy method that provides an important reference for the timely and accurate identification and treatment of PC. Studies to reveal the mechanism of miR‐27a‐5p regulation in PC progression are of great significance to improve the current prognostic status of the tumor.

## 1. Introduction

Parathyroid carcinoma (PC) is a rare endocrine malignancy and is one of the causes of primary hyperparathyroidism (PHPT), accounting for 0.5%–5% of PHPT [1]. PC has a poor prognosis associated with refractory hypercalcemia, which often accompanies advanced PC and is the leading cause of death in patients with this malignancy [2]. One of the factors contributing to this poor prognosis is that PC is not easily differentiated from benign parathyroid adenoma (PA), which results in inaccurate diagnoses and treatment. Recurrent metastasis after surgery leads to uncontrollable hypercalcemia [3]. The second factor contributing to the poor prognosis of PC is the lack of relevant therapeutic options for patients with metastasis and refractory hypercalcemia [4]. This is due to the fact that the mechanisms involved in the development of PC are poorly understood. PC responds poorly to radiotherapy and chemotherapy, and surgery is currently the most effective and reliable treatment for the condition [5]. Therefore, early and timely accurate diagnosis and radical lumpectomy are of great value for preventing metastasis and improving prognosis. In addition, a better understanding of the mechanisms underlying PC progression will facilitate the development of more effective treatments and enhance the accuracy of current diagnostic and therapeutic approaches. However, due to the lack of specific tumor markers, as well as a lack of distinctive clinical symptoms, imaging manifestations, and laboratory tests for PC compared to those for PA, the opportunity for radical surgery is often missed due to misdiagnosis in the preoperative period [6]. Currently, it is extremely challenging to differentiate between PC and PA before surgery using the existing criteria. Furthermore, the proclivity of PC for implantation and metastasis constrains the applicability of pathological procedures such as fine‐needle aspiration of tissues [7]. As a consequence, it is imperative to identify novel molecules associated with PC that differentiate it from PA in liquid biopsies, with the objective of accurately recognizing PC at an early stage and successfully performing primary radical surgery. This lack of understanding of the mechanism of PC progression has led to the current single‐agent treatment, which is unable to cope with highly aggressive tumors that are difficult to completely resect and also foci that appear to have distant metastases. Therefore, there is an urgent need to investigate the relationship between target molecules and tumor progression based on the investigation of PC‐specific molecules related to tumor progression, which will provide a foundation for further studies on potential targeted therapy.

Currently, liquid biopsy has become an important tool for tumor evaluation and is used mainly to detect diagnostic biomarkers that identify early‐stage tumors or recurrent tumors. Many studies have reported the potential of liquid biopsy techniques as diagnostic biomarkers for tumors, including circulating tumor cells (CTCs), circulating tumor DNA (CtDNA), noncoding RNA, and extracellular vesicles (EVs) [8]. Exosomes (exsomes) are lipid bilayer vesicles with a diameter of 40–100 nm that encapsulate proteins, DNA, mRNA, and noncoding RNA and are the primary mediators of cell–cell and cell–microenvironment information exchange [9]. Because the formation of exosomes and the sorting mechanism of their contents are more specific and are relatively stable in the circulation, exosomes have become the most widely regarded class of EVs and play an important role in tumor development, metastasis, and drug resistance [10]. Exosomes secreted by tumor cells contain tumor‐associated specific microRNAs (miRNAs), which are important mediators of tumor stroma‐tumor cell signaling involved in almost all biological processes of tumorigenesis and metastasis [11]. Exosomal miRNAs are also involved in the regulation of several tumors, including lung, prostate, and breast cancers [12–14], and therefore provide a basis for potential diagnosis, prognostic assessment, and therapeutic target exploration of several tumors. In a previous study, we observed differences in miRNA profiles in serum exosomes of PC and PA patients and reported that miR‐27a‐5p differed significantly in serum exosomes of PC and PA patients, suggesting that it may be a potential marker for identifying PC or PA [15]. Functionally, miR‐27a‐5p acts predominantly as an oncomiR, exerting pro‐tumorigenic effects through suppression of several tumor‐suppressor genes. In multiple malignancies, miR‐27a‐5p is consistently upregulated and correlates with higher tumor grade, poor prognosis, and chemoresistance [16]. Experimental silencing of miR‐27a‐5p leads to reduced proliferation and increased apoptosis, supporting its role as a driver of oncogenesis. Nevertheless, due to the limited number of patients with PC, further studies on larger samples are necessary to confirm the findings and to investigate the relationship between exosomal miRNAs and the clinicopathologic features of PCs.

Currently, there is only one clinical treatment for PC, and there are often only a small number of multitargeted antitumor drugs available for advanced patients who have lost the chance of surgery. The therapeutic effect is also not optimistic. The underlying reason is that existing knowledge of PC is relatively limited, especially regarding the basic understanding of the mechanism of PC progression. Despite recent advances in molecular pathology research on PC and PA differential markers, including noncoding RNA, Parafibromin [17], and other agents, very few investigations have been conducted regarding the relationship between target molecules and PC progression and related mechanisms. These studies have indicated that PC progression is associated with alterations in several tumor‐related pathways, including the Wnt pathway [18]. However, studies on the regulatory role of PC‐related molecules on tumor progression at the cellular level and the associated mechanisms have not yet been reported. Furthermore, the impact of the PC‐related exosome, miR‐27a‐5p, on the regulatory relationship with PC cells that we reported previously is also still unknown. Therefore, further investigations are needed urgently to provide a basis for future studies on PC therapeutic targets.

In the current study, we proceeded to analyze the efficacy of exosomal miR‐27a‐5p as a marker for identifying PC or PA and determined its utility for predicting the stage of PC progression using RNA sequencing and RT‐PCR. Concurrently, we also investigated the effect of exosomal miR‐27a‐5p on PC cells and the related target mechanism using the study of PC primary cells.

## 2. Materials and Methods

### 2.1. Patient Enrollment

A total of 65 patients with PHPT who have undergone surgery in our department between 2022 and 2024 were enrolled in the study. Among these, 29 patients had PC and 36 patients had PA. The 36 patients with PA were selected randomly from more than 200 patients with PA who have undergone surgery in our department. The patients included in the study did not suffer from any other endocrine diseases or tumors, and none had received either antineoplastic drugs before surgery or hypocalcemic therapy at the time of peripheral blood specimen collection. The diagnosis of PC was established according to the criteria of the WHO 2022 Parathyroid Tumor Guidelines Consensus, which included the following conditions: (1) vascular invasion, (2) lymphatic invasion, (3) perineural or intraneural invasion, (4) localized malignant invasion of the adjacent anatomical structures, and (5) histologic/cytologic documented metastatic disease. Tumor staging of PC was as follows: T1 tumor, confined to the parathyroid glands, and infiltration limited to soft tissues; T2 tumor, directly invading the thyroid gland; T3 tumor, directly invading the recurrent laryngeal nerve, esophagus, trachea, skeletal muscles, lymph nodes, or thymus; and T4 tumor, directly invading the major blood vessels or spine. Some patients with PC included in the study had lymph node or distant metastases, while some had vascular invasion. We collected clinical information of the patients, including calcium, parathyroid hormone (PTH), and tumor size. Tumor size was defined as the longest diameter of the tumor measured by ultrasound. The study was approved by the Ethics Committee, and written informed consent was obtained from all the patients.

### 2.2. Isolation and Identification of Serum Exosomes

A 3‐mL peripheral blood specimen was obtained from the PC and PA patients prior to surgery and then centrifuged to obtain the serum. For seven PC patients who agreed, we also collected postoperative peripheral blood and obtained the serum. An exosome extraction kit purchased from Umibio was used for the extraction of serum exosomes from the PC and PA patients. The exosomes obtained were dispensed in 50 μL PBS and stored at −80°C. The morphology of the serum exosomes was identified by transmission electron microscopy (TEM) using a volume of 10 μL of exosomes. A 10‐μL aliquot of the sample was aspirated and added dropwise onto a copper grid to precipitate for 1 min, followed by removal of the floating liquid by filter paper aspiration. 10 μL of uranium dioxide acetate was then added to a copper grid and precipitated for 1 min, with the floating liquid removed by filter paper, and the sample was then dried at room temperature for several minutes. A voltage of 100 kV was employed for electron microscopy imaging. The final results of TEM imaging were obtained. Particle size analysis of exosome samples involved 5 μL of exosome solution being diluted to 30 μL and sent for testing. The NanoFCM instrument (Flow Bio Flow NanoAnalyzer) was used to test the particle size and exosome concentration.

### 2.3. MiRNA Sequencing

Five of the 29 patients with PC and 5 of the 36 patients with PA were selected randomly for miRNA sequencing of the serum exosomes. The results obtained were then integrated with those of previously published 4 PC/4 PA exosomal miRNAs. The same method was used for both sequencing tests, followed by analysis of 9 PC/9 PA serum exosomal miRNAs.

The RNA samples were extracted with Trizol, with the purity, concentration, and integrity of the RNA samples tested using advanced molecular biology equipment to ensure the use of qualified samples for transcriptome sequencing. Briefly, ligation of the 3′ SR and 5′ SR adaptors was performed, followed by reverse transcription of the synthetic first chain, and finally, the PCR amplification and size selection. PAGE gels were used for electrophoresis fragment screening purposes, with rubber cutting recycling employed for the small pieces of RNA libraries. The PCR products were then purified (AMPure XP system), followed by assessment of the library quality. The clustering of the index‐coded samples was performed on a cBot cluster generation system using a TruSeq PE Cluster Kit v4‐cBot‐HS (Illumina) according to the manufacturer’s instructions. After cluster generation, the library preparations were sequenced on an Illumina NovaSeq 6000 platform, followed by the generation of single‐end reads.

Data analysis included quality control (QC), comparative analysis, target gene functional annotation, quantification of miRNA expression levels, differential expression analysis, GO enrichment analysis, and KEGG pathway enrichment analysis.

### 2.4. MiRNA Detection

The Trizol method was used to extract miRNAs from the exosomes. The miRNA reverse transcription kit and RT‐PCR kit were obtained from TIANGEN. The reverse transcription system consisted of a 20‐μL system, a reverse transcription program of 42°C for 60 min, 95°C for 3 min, and cDNA storage at −20°C. The primer sequence for miR‐27a‐5p was AGGGCTTAGCTGCTTGTGAG, while the primer sequence for the internal reference U6 was CTCGCTTCGGCAGCACA. The RT‐PCR system was a 20‐μL system, with the following system setup program: The reaction was conducted at 95°C for 15 min, with a denaturation step at 94°C for 20 s and an annealing step at 60°C for 34 s. This sequence was repeated 40 to 45 times. For miRNA quantification, the −ΔCT power of 2 was calculated as relative expression.

### 2.5. Primary Cell Culture and Characterization

Primary cells of PC were obtained by collagenase digestion and differential walling. Collagenase I was purchased from Lamboride. The viable tissue of the excised PC was cut into 1 cm × 1 cm specimens in a sterile environment, placed in EP tubes containing a double‐antibody PBS solution, and transferred to the laboratory on ice. The tissue was rinsed twice with PBS and then cut into crushed tissue pieces, followed by the addition of 10 mL of collagenase I. The tissue was incubated for 40 min in a shaker at 37°C, the supernatant removed by centrifugation at 2000 rpm for 4 min, and the resulting pellet resuspended in PBS. This suspension was then passed through a 100‐μm filter. The plates were resuspended with 1640 medium and transferred to six‐well plates, with the cells cultured overnight at 37°C. The adherent cells were predominantly tumor cells and a few fibroblasts. Cell morphology was observed under a microscope. PC cells were identified by immunofluorescence detection of PTH in PC cells that had been cultured to the 3rd generation. The PTH antibody was purchased from Abcam and was a polyclonal IgG antibody with a murine host. The PC primary cells obtained after isolation can be multiplied and passaged in an average of 3–4 days.

### 2.6. Transfection of Target miRNAs

Lipo3000, purchased from Lamboride, was used for the transfection of primary PC cells. The miR‐27a‐5p mimic was synthesized by Bioengineer. The primary PC cells were transfected on Days 3–4 after wall attachment, with a cell density of 70% considered appropriate. Lipo3000 was dissolved in serum‐free 1640 medium to prepare the transfection solution. The miR‐27a‐5p mimic and NC were also dissolved in serum‐free 1640 medium. The transfection solution was then added to the culture wells, mixed, and incubated at 37°C for 48 h. Following this incubation period, the cells were digested and harvested to obtain RNA. The transfection efficiency was then detected by RT‐PCR. In the exosome mixing experiments, we added 50 ul of previously obtained PC exosomes as well as 50 µl of PBS as a control to two groups of PC cell cultures, respectively.

### 2.7. Apoptosis Function Assay

The cells were digested and stained using the Annexin V‐PITC reagent purchased from Lamboride. Apoptosis detection was performed by on‐board flow cytometry.

### 2.8. Dual Luciferase Assay

Construction of the h‐PIK3CA vector was carried out by Hanheng Biotechnology Co. The extracted plasmids were used to transfect the cells after QC validation. The 293T cells cultured in DMEM and the target plasmid were used for transfection. The h‐PIK3CA‐3UTR target plasmid and the miR‐27a‐5p/negative control (NC) were dissolved in DMEM and mixed thoroughly, with the cells then transfected with the transfection reagent dissolved in DMEM. The transfection reagent was purchased from Hanheng Biotechnology and had a concentration of 0.8 mg/mL. Subsequently, the assay was conducted using the dual‐luciferase reporter assay kit (Hanheng Bio Products). Primary cell experiments and dual luciferase experiments were performed in three biological replicates.

### 2.9. Statistical Analysis

All statistical analyses were conducted using GraphPad Prism 8.0 and SPSS 22.0. Continuous variables were analyzed using the Mann–Whitney *U* test, while categorical variables were analyzed using the *χ*2 test. Statistical significance was determined by a *p* value of < 0.05. The diagnostic efficacy was evaluated by plotting the subject’s work characteristics (ROC) curve, with sensitivity as the vertical coordinate and 1‐specificity as the horizontal coordinate. Spearman’s correlation analysis was used for the correlation analysis.

## 3. Results

### 3.1. Patient Information and Clinicopathologic Characteristics

Table 1 presents the basic information of the 65 patients with PHPT. Of the 29 patients with PC, 15 were male and 14 were female, with a median age of 55 years. Of the 36 patients with PA, 3 were male and 33 were female, with a median age of 49.5 years. There were statistically significant differences in the levels of blood calcium (*p* = 0.0193) and PTH (*p* = 0.0179) between the two patient groups. Calcium and PTH levels were higher in the PC group. There was also a statistically significant difference in tumor size between the two groups (*p* = 0.0037). There were no significant differences in phosphorus (*p* = 0.0629) and vitamin D (*p* = 0.7606) levels between the two groups. Of the 29 patients with PC, 22 cases had T1‐T2 staging and 7 cases had T3–T4 staging, with 8 cases of vascular invasion in their pathological tissue specimens. Lymph node metastasis was present in 6 cases, and distant metastasis was present in 7 cases.

**TABLE 1:** Clinicopathological characteristics of PC and PA groups.

Characteristic	PC (*n* = 29)	PA (*n* = 36)	*p* value
Gender, *n* (%)			0.0001^a^
Male	15 (51.7%)	3 (8.3%)	
Female	14 (48.3%)	33 (91.7%)	
Age (years)	55.5	49.5	0.5352^b^
Tumor size (cm)	2.37 ± 1.23	1.52 ± 0.72	0.0037^b^
Calcium (mmol/L)	2.95 ± 0.48	2.73 ± 0.21	0.0193^b^
Phosphorus (mmol/L)	0.80 ± 0.27	0.91 ± 0.17	0.0629^b^
PTH (pg/mL)	479.34 ± 555.47	252.66 ± 341.27	0.0179^b^
Vitamin D (ng/mL)	16.24 ± 8.02	16.77 ± 8.12	0.7606^b^
T stage			
T1‐T2	24 (82.8%)		
T3‐T4	5 (17.2%)		
Vascular invasion			
Yes	8 (27.6%)		
No	21 (72.4%)		
Lymph node metastasis			
Yes	6 (20.7%)		
No	23 (79.3%)		
Distant metastasis			
Yes	7 (24.1%)		
No	22 (75.9%)		

*Note:* Data are presented as the means ± SD.

^a^Analyzed using chi‐square test.

^b^Analyzed using Mann–Whitney test.

### 3.2. Sequencing Analysis of Serum Exosomal miRNAs From PC/PA Patients Showed Significant Differences in miR‐27a‐5p

The acquired patient serum exosomes were analyzed and identified by TEM and particle size analysis. TEM demonstrated that the serum‐isolated exosomes exhibited a typical circular or elliptical structure, with diameters ranging from 40 to 150 nm (Figure 1A, B). In addition, particle size analysis showed that the majority of the exosomes had diameters within the range of 40–100 nm (Figure 1C). Exosomal miRNA sequencing of 9 PC/9 PA detected 4837 miRNAs, including 1742 known miRNAs and 3095 newly predicted miRNAs. Differential expression analysis screening of a number of miRNAs showed significant differences (*p* < 0.05 and > 2‐fold change), including miR‐27a‐5p, which we have reported previously (Figure 1D, E) [15]. We then performed differential miRNA target gene prediction and target gene enrichment using the GO/KEGG database analysis in order to investigate the possible mechanistic pathways associated with PC. A total of 20 predicted signaling pathways were listed. Among these, the Focal adhesion, MAPK, and Wnt pathways appeared to be involved mainly in the function of PC exosomal miRNAs (Figure 1F, G). These pathways have been shown to be associated with the development of a variety of cancers.

FIGURE 1Exosome identification and exosomal miRNA sequencing analysis. (A, B) Transmission electron microscopy observation of exosome morphology. (C) Exosome particle size analysis. (D) Volcano map of differential miRNAs obtained by PC/PA serum exosomal miRNA sequencing. (E) Heat map of differential miRNAs obtained by PC/PA serum exosomal miRNA sequencing. (F) Differential miRNA target gene enrichment by GO database analysis. (G) Differential miRNA target gene enrichment obtained by KEGG database analysis.

**Figure:**
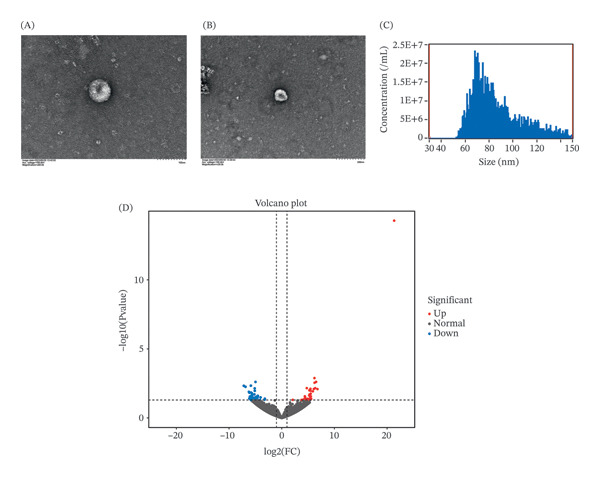

**Figure:**
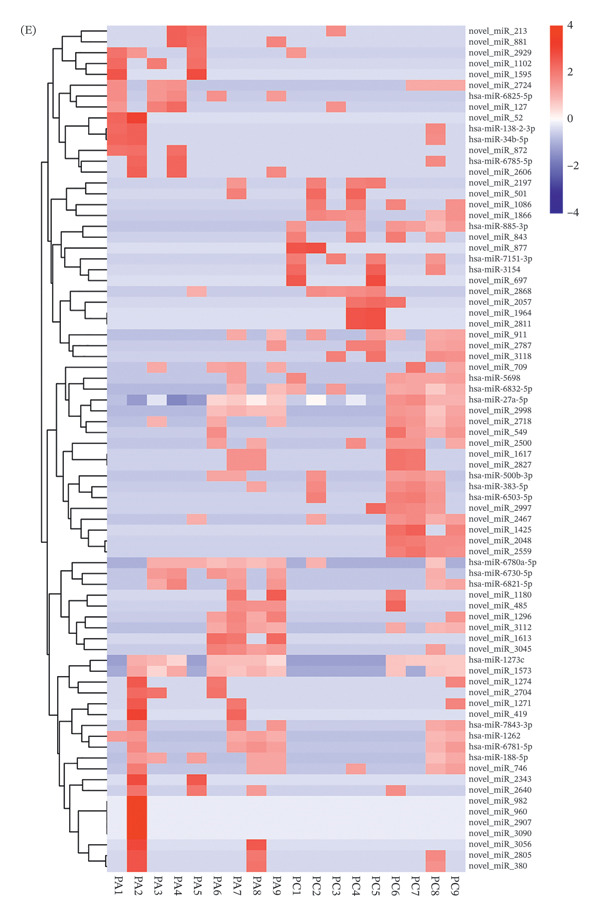

**Figure:**
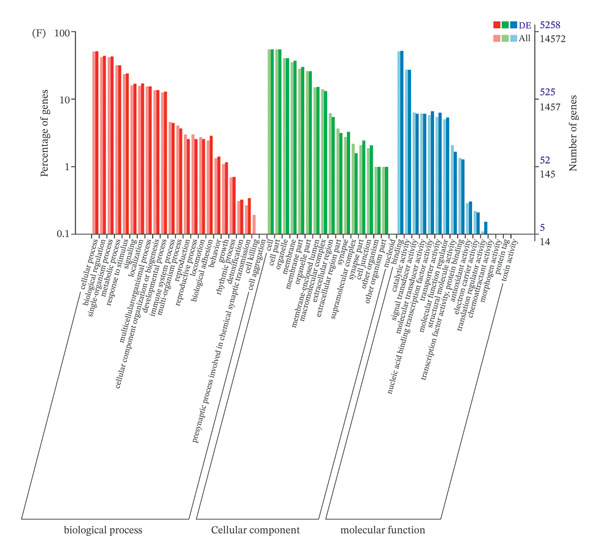

**Figure:**
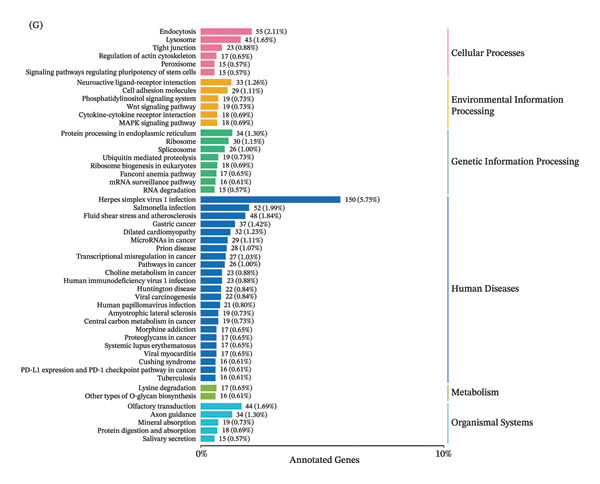

### 3.3. Differential Expression of Exosomal miR‐27a‐5p in PC/PA, Analysis of Diagnostic Efficacy, and Relationship With the Clinicopathologic Features of PC

**FIGURE 2:**
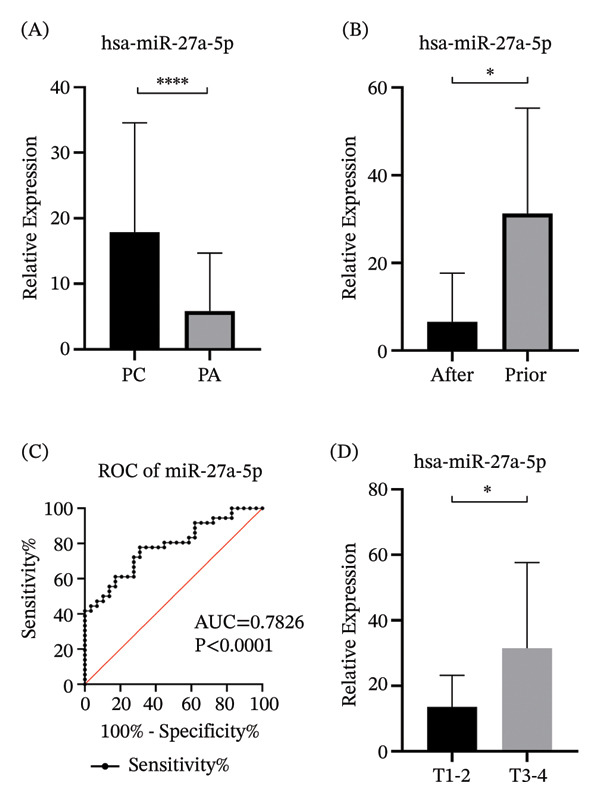
Differential expression analysis of exosomal miR‐27a‐5p in PC/PA and its association with clinicopathological features of PC. (A) The difference of exosomal miR‐27a‐5p in PC/PA peripheral blood was significant. (B) Serum exosomal miR‐27a‐5p was decreased significantly in PC patients after tumor resection, suggesting that exosomal miR‐27a‐5p was mainly of tumor origin. (C) The diagnostic efficacy of exosomal miR‐27a‐5p for identifying PC/PA was good, with an AUC of 0.7826. (D) Exosomal miR‐27a‐5p correlated with the T stage of PC tumors, with the exosomal miR‐27a‐5p of T3‐T4 tumors in the progressive stage being significantly higher than that of T1‐T2 tumors in the early stage.

RT‐PCR was used to detect the differential expression of serum exosome miR‐27a‐5p in 29 PC and 36 PA patients. The results demonstrated that miR‐27a‐5p differed significantly in PC and PA (*p* < 0.0001; Figure 2A). Furthermore, serum exosome miR‐27a‐5p was downregulated significantly in seven cases of PC tumor resection compared with that observed before tumor resection (*p* = 0.0469; Fig 2B). These findings suggest that serum exosomal miR‐27a‐5p may originate from PC tumors or alternatively be closely associated with the presence of tumors. A diagnostic efficacy analysis showed that the area under the curve (AUC) of exosomal miR‐27a‐5p was 0.7826 (95% CI, 0.6731–0.8920; Figure 2C). With regard to clinical biochemical markers, there were no significant differences in phosphorus and vitamin D levels between PC/PA. While the AUC of calcium was 0.6691, the AUC of PTH was 0.6710. Our data indicated that exosomal miR‐27a‐5p may be a more effective means of differentiating PC from PA. We further investigated the relationship between exosomal miR‐27a‐5p and clinicopathologic characteristics of patients with PC. For continuous variables, such as preoperative calcium and PTH levels, statistical analysis showed that exosomal miR‐27a‐5p did not correlate significantly with any of these variables. Furthermore, there was no significant correlation between exosomal miR‐27a‐5p and tumor size. For PCs with different pathological features, the results demonstrated that there was no statistically significant difference in exosomal miR‐27a‐5p between lymph node metastasis‐negative patients and lymph node metastasis‐positive patients. There were also no significant differences in exosomal miR‐27a‐5p between patients with or without distant metastasis or with or without vascular invasion. In contrast, there was a correlation between PC tumor T‐stage and exosome miR‐27a‐5p. Specifically, PCs with T1‐T2 exhibited significantly different exosomal miR‐27a‐5p compared to PCs with T3–T4 (Figure 2D).

### 3.4. Exosomal miR‐27a‐5p Affects PC Cell Apoptosis

**FIGURE 3:**
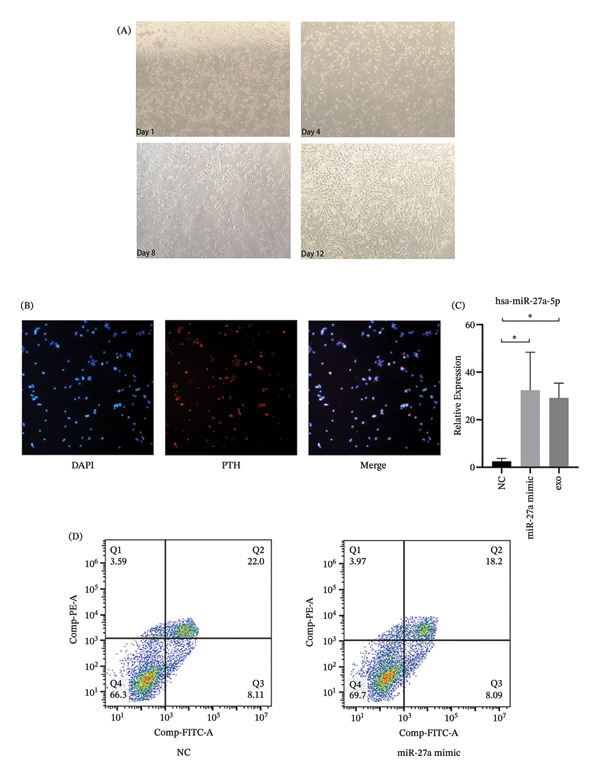
Identification of primary PC cell culture and the effect of exosomal miR‐27a‐5p on PC cell apoptosis. (A) Microscopic observation of PC progenitor cells. (B) Immunofluorescence identification of PC progenitor cells, PTH (+). (C) Verification of transfection efficiency: miR‐27a‐5p expression was upregulated significantly in PC progenitor cells after miR‐27a‐5p mimic transfection, and was also upregulated significantly after mixing and culturing with PC serum exosomes. (D) Apoptosis of PC cells after miR‐27a‐5p mimic transfection was inhibited.

Primary cells of PC were obtained by collagenase digestion and differential walling. After performing passaging, PC cells with homogeneous morphology, which were classically rounded, were still observed under the microscope (Figure 3A). Immunofluorescence demonstrated positive PTH staining, which could elucidate the subjectivity of the PC cells (Figure 3B). After transfection with miR‐27a‐5p mimic or a mixed culture of PC serum exosomes, miR‐27a‐5p expression was upregulated in both PC cells (Figure 3C). Subsequent flow cytometry results demonstrated that after transfection with the miR‐27a‐5p mimic, PC cell apoptosis was inhibited significantly, suggesting that miR‐27a‐5p inhibited PC cell apoptosis (Figure 3D). However, PC cell apoptosis was not obviously inhibited after mixed culture with PC serum exosomes (Figure S1).

### 3.5. PIK3CA Is a Target Gene of miR‐27a‐5p and May Be an Important Target for Its Regulation of PC Cell Apoptosis

**FIGURE 4:**
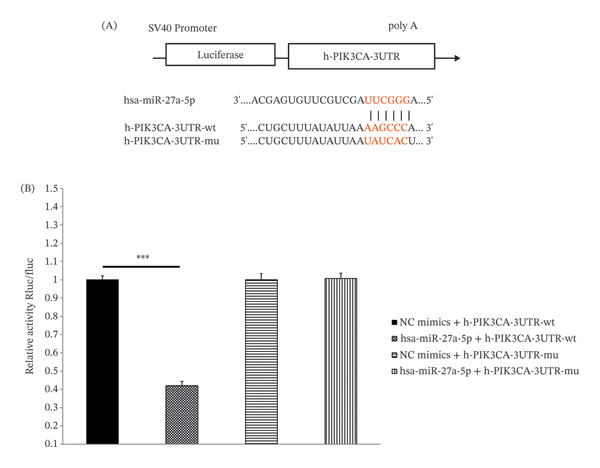
miR‐27a‐5p target gene prediction and dual luciferase validation. (A) The presence of the PIK3CA binding arm of miR‐27a‐5p was demonstrated by target gene prediction. (B) A dual luciferase assay showed that miR‐27a‐5p significantly downregulated the expression of luciferase in h‐PIK3CA‐3UTR‐WT (*p* < 0.001), whereas miR‐27a‐5p failed to downregulate the expression of h‐PIK3CA‐3UTR‐MUT. This indicated that there is a binding effect between the two in this experiment and that PIK3CA is the target gene of miR‐27a‐5p.

We demonstrated the presence of a binding site for PIK3CA in miR‐27a‐5p using target gene prediction of miR‐27a‐5p via the miRDB website (Figure 4A). This suggested that PIK3CA may act as a target gene of miR‐27a‐5p. This is an important initiator gene of the PI3K‐AKT pathway, with activation of the pathway closely related to tumor apoptosis. We postulated that the mechanism by which miR‐27a‐5p regulates apoptosis in PC cells may be related to its target gene PIK3CA. Further dual luciferase experiments demonstrated that miR‐27a‐5p significantly reduced the expression of luciferase in h‐PIK3CA‐3UTR‐WT in comparison with that observed in the NC group (*p* < 0.001). This finding indicated a binding effect between the two in this experiment and that PIK3CA was the target gene of miR‐27a‐5p. In experiments with mutant phenotypes, miR‐27a‐5p was unable to downregulate the expression of luciferase in h‐PIK3CA‐3UTR‐MUT compared with that observed in the NC group (*p* > 0.05; Figure 4B). On the basis of these findings, we concluded that PIK3CA is a target gene of miR‐27a‐5p and may be an important target for its regulation of PC cell apoptosis. However, the specific mechanism of this interaction remains to be elucidated.

## 4. Discussion

The current state of PC treatment is very disappointing, with a significant number of PC patients still dying from uncontrolled hypercalcemia due to recurrent tumors [19]. Implantation metastasis represents the primary factor in the postoperative recurrence of PC. The key factors contributing to this phenomenon are the difficulties of recognizing PC and performing radical surgery in a timely and precise manner [20]. Currently, the diagnosis of PC is primarily based on limited histopathological features observed during or after surgery, such as envelope invasion, vascular invasion, and other traditional pathological methods. These methods rely on the metastasis that has occurred to diagnose malignant tumors, and although highly accurate, they may result in the miss of the optimal time for treatment, sometimes at the expense of the patient’s life [21]. The existing preoperative laboratory tests and imaging examinations are unable to distinguish PC from PA. As a consequence, PC is often misdiagnosed as PA without radical surgery being performed. This “preoperative misdiagnosis—implantation metastasis—recurrent hypercalcemia” is a common poor regression pattern of PC [22]. As a result, there is a pressing need for the development of a liquid biopsy molecular marker that can effectively differentiate PC from PA at an early stage, with the aim of improving the current poor prognosis of PC. Exosomal miR‐27a‐5p not only has the potential to better distinguish between PC and PA, but also has good stability and has become an established detection method. Among the various liquid biopsy methods, ctDNA is susceptible to interference, CTC detection is limited by the expression of its epitope and lacks specificity, while the stability of noncoding RNA detection is poor. In contrast, exosomal miRNA combines the strength of specificity, stability, and easy detection, thereby offering a significant advantage over other liquid biopsy methods. Furthermore, for recurrent patients who have lost the opportunity for surgical intervention, the availability of alternative therapeutic options remains limited, while the efficacy of hypocalcemic therapy for hypercalcemia is suboptimal. Therefore, there is a pressing need to identify and develop targeted therapies that improve the current treatment options. To this end, it is imperative to investigate the role and mechanisms by which PC‐related molecules regulate their progression.

Exosomal miRNAs have been demonstrated to be valuable for diagnosing tumors such as lung, breast, and thyroid cancer [23–25]. For PC, it has been reported that miR‐139, miR‐30b, and miR‐126 are differentially down‐regulated in PC and PA tissues [26]. These miRNAs have been identified as potential molecular markers for PC. Our previous study was the first to identify a differential miRNA profile of PC and PA serum exosomes and demonstrated that exosomal miR‐27a‐5p exhibited differential significance between PC and PA, despite the specimen volume being relatively limited. Incorporating miR‐27a‐5p into diagnostic workflows offers several advantages: (1) objective molecular readout; (2) complementary biomarker with parafibromin and CDC73 status; and (3) potential for noninvasive testing. The main limitation remains the need for standardized cutoff values and multicenter validation.

In the current study, we demonstrated that exosomal miR‐27a‐5p exhibited significant differences between PC and PA, with high diagnostic efficacy. Our findings were validated through large‐sample sequencing and assay analysis. When exosomal miR‐27a‐5p had high relative expression, we observed a heightened probability of PC, which indicated radical surgery with en bloc resection might be necessary. miR‐27a‐5p demonstrates superior diagnostic performance for PC/PA compared to biochemical markers such as calcium and PTH. PC‐specific molecular diagnostic efficacy studies in the same field showed that the AUC of exosomal miR‐27a‐5p was at a high level. In comparison to other PC and PA differential markers, exosomal miRNAs are distinguished by high stability and good specificity, but require more advanced technology for their detection. A multitude of studies have reported PC‐specific pathologic markers [27]. However, early and precise diagnosis and treatment are more likely to improve PC prognosis than that achieved by remediation after definitive pathology is observed. However, there is a paucity of studies on liquid biopsy and PC. Therefore, it is feasible and important to develop a novel scoring system to distinguish between PC and PA. This system would be beneficial for individualized assessment of the possibility of PC, reducing misdiagnosis and omission of diagnosis, guiding the timely diagnosis of PC at an early stage, and indicating the need for radical surgery. Such a system would therefore be of great value for reducing the recurrence and metastasis of PC, thereby improving prognosis.

To investigate the potential of exosomal miRNA to predict the progression and staging of PC, we examined the relationship between clinicopathologic features of PC and exosomal miR‐27a‐5p. The results demonstrated that exosomal miR‐27a‐5p was not significantly associated with the pathological features of PC, such as lymph node metastasis, distant metastasis, and vascular invasion. However, exosomal miR‐27a‐5p correlated with the T stage of the tumor, suggesting that it has the potential to be used as a marker for predicting the tumor stage of PC. We observed no correlation between exosomal miR‐27a‐5p and examination indices, including PTH and blood calcium levels, and tumor size. Previous studies have demonstrated a close association between exosomal miRNA and the pathological features and staging of lung, bladder, and esophageal cancers, among other malignancies [28–30]. However, we were unable to obtain relevant and meaningful conclusions in our assessment of the relationship between the clinicopathological features of PC and exosomal miR‐27a‐5p. This may be due to limitations in the sample size, which made it difficult to obtain a statistically significant difference. However, the results of our study indicated that exosomal miR‐27a‐5p was associated with PC tumor stage, a finding that suggests that miR‐27a‐5p may serve as a potential marker to guide PC progression and risk level assessment.

Few studies have examined the role and mechanism of PC‐related molecules in regulating the tumor’s effects, mainly due to the rarity of PC and the lack of corresponding cell lines. Some studies have identified primary PC cells in culture [31], while experimental exploration of molecular functions at the cellular level is still insufficient. Like most other malignant tumors, the development of PC is a very complex process involving multiple signaling pathways and different mechanisms [32]. Sequencing analysis in the current study showed that important mechanistic pathway changes associated with PC may be related to tumor progression. Target gene prediction showed that PIK3CA, the target gene of miR‐27a‐5p, is an important initiator gene of the apoptosis‐related PI3K‐AKT pathway. In this study, following the acquisition of PC primary cells and successful passaging culture, we first investigated the effect of exosomal miRNA on PC cells. This involved characterizing primary PC cells obtained by a differential apposition method after passaging, with the results showing that upregulation of miR‐27a‐5p expression inhibited PC cell apoptosis. This result provided an important theoretical basis for the study of exosomal miR‐27a‐5p as a PC tumor marker and future target therapy. Other studies have shown that exosomal miR‐27a‐5p regulates tumor cell apoptosis in thyroid cancer [33], and we reached a similar conclusion in PC cells. However, the apoptotic function of PC cells after mixed culture with PC serum exosomes was not affected significantly, despite upregulation of miR‐27a‐5p expression. This might be due to the fact that PC cells may be regulated by carrying target miRNAs in exosomes, despite these exosomes possibly also containing other molecular components that affect the biological behavior of tumors. Another earlier study also reported that activation of the PI3K‐AKT signaling pathway may be associated with PC pathogenesis and progression [34]. Our subsequent study showed that PIK3CA served as a target gene for miR‐27a‐5p, and that miR‐27a‐5p inhibited PC cell apoptosis, possibly related to its target gene PIK3CA. This sequence of events provided a theoretical basis for future molecularly targeted therapy of PC. However, further studies are required to elucidate the precise molecular mechanism by which exosomal miR‐27a‐5p inhibits tumor cell apoptosis in PC. Currently, the study of exosomal miRNA is increasing, while the technology of isolation and purification of exosomes in serum is also developing. With the ongoing development of this research, exosomal miRNAs will have broader application and potential in tumor diagnosis and treatment.

This study has some limitations. First, the study did not investigate the predictive value of exosomal miR‐27a‐5p on PC prognosis, which requires further in‐depth investigation in long‐term follow‐up studies. Second, the study did not elucidate the detailed mechanism by which exosomal miR‐27a‐5p targets PIK3CA to inhibit tumor cell apoptosis. Finally, due to the rarity of PC and the lack of corresponding cell lines, the sample size of this study was limited at both the clinical and cellular levels. To further validate the findings of this study, multicenter and large‐sample studies are needed in the future.

In conclusion, exosomal miR‐27a‐5p identified significant differences between PC and PA, making it a reliable indicator for identifying PC and an important reference for timely and accurate PC treatment. In the future, precise differentiation between PC and PA combined with radical surgery based on exosomal miR‐27a‐5p‐related results will be of great significance for improving the current status of PC diagnosis and treatment and enhancing prognosis. Our study also demonstrated that exosomal miR‐27a‐5p targets PIK3CA, thereby inhibiting tumor cell apoptosis. Taken together, these results provide a theoretical basis for understanding the mechanisms of PC progression at the cellular and molecular levels, as well as for developing future PC‐related molecular targeted therapies. The results also offer cutting‐edge therapeutic ideas and strategies for the diagnosis and treatment of PC.

## Author Contributions

W.J. and W.Y. were the co‐first authors. W.J. contributed to the conception of the study. W.Q. and S.H. designed the experiments. Z.T. and L.X. performed the material preparation. W.Y., H.J., and Y.W. performed data collection. W.J. and F.D. performed the formal analysis. W.J. and W.Y. wrote the original draft of the manuscript. S.R., Z.L., and W.B. commented on previous versions of the manuscript.

## Funding

This research received no external funding.

## Disclosure

All authors read and approved the final manuscript.

## Ethics Statement

The study was conducted according to the guidelines of the Declaration of Helsinki and approved by the Ethics Committee of Beijing Chaoyang Hospital, Capital Medical University (approval number 2022‐Ke‐282).

## Conflicts of Interest

The authors declare no conflicts of interest.

## Supporting Information

Additional supporting information can be found online in the Supporting Information section.

## Supporting information


**Supporting Information** Fig. S1. Apoptosis of PC cells mixed with PC exosomes: PC cell apoptosis was not obviously inhibited after mixed culture with PC serum exosomes.

## Data Availability

The datasets generated during and/or analyzed during the current study are available from the corresponding author on reasonable request.
