# Supplementary figures and images for: Exosomal miR‐27a‐5p Helps Differentiate Parathyroid Carcinoma From Adenoma and Inhibits Apoptosis in Parathyroid Carcinoma Cells
Source: Int J Endocrinol. 2026 Jun 8;2026:5562632. doi: 10.1155/ije/5562632 (PMC13244254; doi:10.1155/ije/5562632)

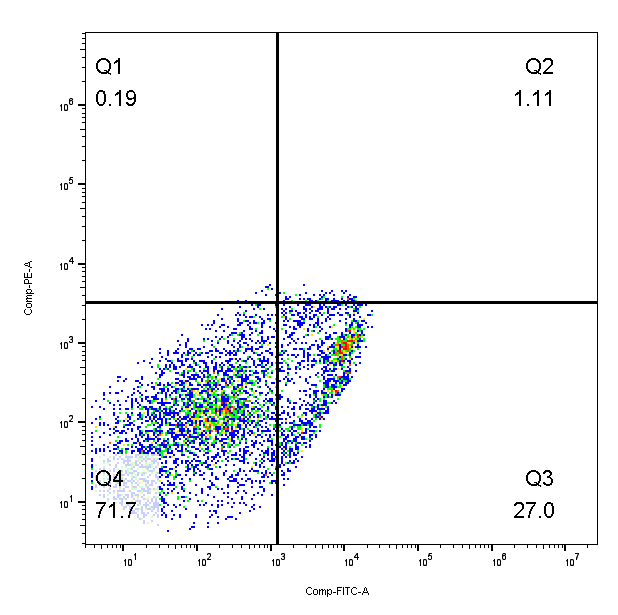

Supplement: Supplementary file 1 — Supporting Information Fig. S1. Apoptosis of PC cells mixed with PC exosomes: PC cell apoptosis was not obviously inhibited after mixed culture with PC serum exosomes. [file IJE-2026-5562632-s001.png]
